# Reducing youth suicide: systems modelling and simulation to guide targeted investments across the determinants

**DOI:** 10.1186/s12916-021-01935-4

**Published:** 2021-03-12

**Authors:** Jo-An Occhipinti, Adam Skinner, Frank Iorfino, Kenny Lawson, Julie Sturgess, Warren Burgess, Tracey Davenport, Danica Hudson, Ian Hickie

**Affiliations:** 1 Brain and Mind Centre, Faculty of Medicine and Health, University of Sydney, Camperdown, Australia; 2 Computer Simulation & Advanced Research Technologies (CSART), Sydney, Australia; 3 Menzies Centre for Health Policy, University of Sydney, Sydney, Australia; 4 Translational Health Research Institute, Western Sydney University, Penrith, Australia; 5 Hunter Medical Research Institute, Newcastle, Australia; 6 North Coast Primary Health Network, Ballina, Australia

**Keywords:** Suicide prevention, Strategic planning, Decision analysis, Systems modelling, Simulation

## Abstract

**Background:**

Reducing suicidal behaviour (SB) is a critical public health issue globally. The complex interplay of social determinants, service system factors, population demographics, and behavioural dynamics makes it extraordinarily difficult for decision makers to determine the nature and balance of investments required to have the greatest impacts on SB. Real-world experimentation to establish the optimal targeting, timing, scale, frequency, and intensity of investments required across the determinants is unfeasible. Therefore, this study harnesses systems modelling and simulation to guide population-level decision making that represent best strategic allocation of limited resources.

**Methods:**

Using a participatory approach, and informed by a range of national, state, and local datasets, a system dynamics model was developed, tested, and validated for a regional population catchment. The model incorporated defined pathways from social determinants of mental health to psychological distress, mental health care, and SB. Intervention scenarios were investigated to forecast their impact on SB over a 20-year period.

**Results:**

A combination of social connectedness programs, technology-enabled coordinated care, post-attempt assertive aftercare, reductions in childhood adversity, and increasing youth employment projected the greatest impacts on SB, particularly in a youth population, reducing self-harm hospitalisations (suicide attempts) by 28.5% (95% interval 26.3–30.8%) and suicide deaths by 29.3% (95% interval 27.1–31.5%). Introducing additional interventions beyond the best performing suite of interventions produced only marginal improvement in population level impacts, highlighting that ‘more is not necessarily better.’

**Conclusion:**

Results indicate that targeted investments in addressing the social determinants and in mental health services provides the best opportunity to reduce SB and suicide. Systems modelling and simulation offers a robust approach to leveraging best available research, data, and expert knowledge in a way that helps decision makers respond to the unique characteristics and drivers of SB in their catchments and more effectively focus limited health resources.

**Supplementary Information:**

The online version contains supplementary material available at 10.1186/s12916-021-01935-4.

## Background

Reducing suicidal behaviour (suicide attempts and deaths) remains a critical public health issue globally. Over the period 1990 to 2016, suicide was estimated to be the leading cause of age-standardised years of life lost in high-income Asia Pacific countries, and among the top 10 leading causes in Europe, and parts of the Americas [1, 2]. The World Health Organization’s (WHO) development of a Comprehensive Mental Health Action Plan [3] and the inclusion of suicide mortality as an indicator for the Sustainable Development Goals (SDGs) [4] have signalled a recognition of the role mental health, mental capital, and suicidal behaviour (SB) play in social, cultural, and economic participation that contributes to the mental wealth of nations [5, 6] and in facilitating or undermining progress towards broader international development targets [7]. Further significant momentum has been achieved through the World Economic Forum’s Global Shapers Community, a grassroots network of young people, who called on all countries to increase financing, public mental health education, and quality systems of care at the Annual meeting in Davos in 2020 [8]. This elevation of mental health and wellbeing and suicide prevention in the global development agenda has led to a renewed push worldwide to strengthen mental health systems (particularly leadership and governance, and community-based care), increase service coverage and responsiveness, and set targets for reductions in suicide deaths [3, 9, 10].

Despite this momentum, growing evidence of effective suicide prevention interventions, and the release of successive action plans, current strategies are not delivering substantial impacts [11–13]. SB has a complex aetiology with a wide range of contributing factors, both individual and contextual, and is rarely a result of any single cause [14, 15]. While there is good evidence for the significant role mental ill-health has in the aetiology of suicide [16–18], discourse has turned to the role of social determinants in the causal mechanism of mental disorder and SB as targets for prevention [19–21]. Factors including adverse childhood exposures, domestic and family violence, substance abuse, unemployment, and other socioeconomic factors that influence access to housing and mental health services have been found to have unidirectional or bidirectional relationships with each other and with psychological distress, mental disorder, and suicide [22–27]. However, the relationship of these risk factors with mental health and suicide outcomes are often analysed using methods that assume they are independent and that their relationship with key outcomes are linear and constant through time [28], and as such, interventions to address these risk factors are explored discretely. The complex interplay of social determinants, service system factors, population demographics, and behavioural dynamics makes it extraordinarily difficult for decision makers to determine the nature and balance of investments required to have the greatest impacts on suicidal behaviour over the short and long term. While there are numerous evidence reviews to support the case for investments aimed at addressing the social determinants of mental disorder, it is unclear whether resources should be spread across each of them or whether some are more important than others for suicide prevention in a particular context.

Systems modelling and simulation offers an important tool for systems analysis to support decision making for complex problems. Systems modelling is a robust quantitative method of complex systems science, an interdisciplinary field that studies the nature and behaviour of complex systems underpinned by well-established mathematical theory of nonlinear dynamics [29–33]. It provides a robust method for mapping and quantifying the complex causal mechanism driving mental health and suicide outcomes [11, 34–36]. Systems modelling is uniquely able to capture population and demographic dynamics, changes over time in social and economic drivers of psychological distress, mental disorders and suicidal behaviours (including feedback loops), workforce dynamics and the changing relationship between service supply versus demand, and the potentially non-additive (interdependencies and interacting) effects of intervention combinations, factors that bedevil traditional analytic approaches. Model development leverages disparate datasets, research evidence, and our best understanding of local system structure and behaviours of system actors in a systematic and disciplined way [28, 37–42]. The process delivers an interactive decision support tool that provides a virtual environment to explore the optimal combination, targeting, timing, scale, frequency, and intensity of investments in screening, treatment, population-based mental health strategies, and social determinants required to achieve the greatest impacts within the contextual, resource, and capacity constraints of a particular region, before implementing them in the real world.

This study describes the application of systems modelling and simulation undertaken as a research-practice partnership between a regional Primary Health Network (PHN) in New South Wales, Australia, their stakeholders, and several academic institutions. The study aimed to leverage a range of national, state, and local datasets to (i) identify the likely impact over time of a range of locally prioritised mental health and suicide prevention interventions being considered for investment, (ii) determine the value and balance of investments across the social determinants of mental health in the region, and (iii) determine the best combination of strategies to deliver the greatest impacts on suicidal behaviour.

## Methods

### Context

The North Coast PHN supports a population of 502,524 (as at 2016) [43], distributed over a geographic area of approximately 35,570 km^2^ and taking in both coastal and inland rural communities. Aboriginal and/or Torres Strait Islander people represent approximately 5.0% of the North Coast population, which is higher than the proportion for both New South Wales (2.9%) and Australia (2.6%) [44]. The region has a high proportion of the population aged over 65 years (20.4%), and is more socio-economically disadvantaged than the national average [44]. Unemployment is high across the North Coast region, with some local government areas (LGAs) reporting rates as high as 9.7% compared to the national average of 5.9% in 2018 [44]. Domestic and family violence rates are higher than the NSW average, with some LGAs reporting incidence of domestic assaults as high as 757.1 per 100,000 population [45], as are rates of homelessness, with some LGAs reporting rates as high as 57.5 per 10,000 population [46]. Rates of suicide are also well above the NSW average, with incidence rising from 7.9 per 100,000 in 2006 to 16.6 per 100,000 population in 2017 [47]. As of mid-2016, young people aged 15–24 years made up 10.4% of the north Coast PHN population, which was lower than the proportions of young people in the NSW population (13.9%) and the Australian population (13.2%). In 2016–2017, the rate of children and young people being in out of home care was higher in the North Coast PHN region (16.3 per 1000 children aged 0–17 years) compared to 11.4 per 1000 children in NSW [44]. The rate of children and young people reported as being at risk of significant harm was also higher in the region (79.7 per 1000 children aged 0–17 years) compared to 52.3 per 1000 children in NSW [44].

### Model development

A system dynamics model was developed using a participatory modelling approach that involved approximately 50 local stakeholders, including representatives from health and social policy agencies, non-government organisations, primary care providers, emergency services, research institutions, community groups, and, importantly, people with lived experience of suicide. The process employed a broad systems perspective drawing on the deep tacit knowledge and diverse perspectives of these system actors. Input from stakeholders was provided through a series of workshops, meetings, priority setting surveys and system mapping activities conducted in 2019 (see video: http://nccforbetterlives.com.au/systems-modelling). The participatory modelling process undertaken for the current study followed the approach detailed elsewhere [48–50]. In summary, workshop 1 (full day) included discussion and prioritisation of the key outcomes of interest, the mapping of pathways and drivers of those outcomes, and the prioritisation of interventions to be included in the model. Following the workshop, the map was converted to a conceptual model which was synthesised with best available research evidence and data to inform the development of the initial system dynamics model. In addition, a draft structure representing the service pathways of the local mental health system developed from workshop 1 was disseminated to broader community stakeholders for verification and input. Stakeholders returned their modifications to the draft structure, and feedback from this process was synthesised with missing pathways subsequently integrated into the system dynamics model. Several months after workshop 1, the 50 stakeholders reunited for workshop 2 (full day) where the draft structure, logic, and key assumptions of the model were presented for verification, discussion, feedback, and consensus. In addition, participants were supported to map the mechanisms of effect of prioritised interventions (priorities determined via survey) onto the structure of the system dynamics model. Reviews of the literature following workshop 2 further informed intervention mechanisms and parameters, and an interactive interface was developed. A final (half-day) workshop 3 was conducted several months after workshop 2 where the penultimate version of the model was presented to the stakeholders for verification, discussion, feedback, and consensus. Stakeholders were provided with the opportunity to interact directly with the model interface to run scenarios, test alternative assumptions, discuss and question results, and provide feedback on interface design and functionality. The key insights derived from the model and their implications for service planning, commissioning of programs and services, and evaluation were presented and discussed to ensure face validity.

Model development does not follow a linear process; it is an iterative knowledge feedback process that entails continuous hypothesis development, testing, and refinement—a process shared with the multidisciplinary group both through the workshops and additional out-of-session meetings as required with stakeholder sub-groups. Model structure, parameter estimates, and other numerical inputs were informed (where possible) by published research or available regional, state, and national data or were estimated via constrained optimisation (see Additional file 1). Model construction and analysis were performed using Stella Architect ver. 1.9.4 (www.iseesystems.com). The model was validated by (i) testing whether the model could replicate historic data across a range of key indicators (namely; time series of psychological distress, psychiatric hospitalisations, emergency department (ED) presentations, youth and total population self-harm hospitalisations, and suicide deaths—see Additional file 1: Fig. S5, Fig. S7, Fig. S17, and Fig. S22) and (ii) ensuring face validity of the model structure and performance among stakeholders working in or interacting with different parts of the system.

### Model structure, outputs, and calibration

The core model structure included (1) a population component, capturing changes over time in the size and composition of the population resulting from births, migration, ageing, and mortality; (2) a psychological distress component that models flows of people to and from states of low psychological distress (Kessler 10 [K10] score 10–15), moderate psychological distress (K10 score 16–21), and high to very high psychological distress (K10 score 22–50); (3) a series of components capturing the interdependent dynamics of key social determinants, namely, early life exposures, substance abuse, domestic violence, homelessness, and unemployment, and their influence on levels of psychological distress; (4) a mental health services component that models the movement of psychologically distressed people through one of several possible service pathways involving (potentially) general practitioners, psychiatrists and allied mental health professionals (including psychologists and mental health nurses), emergency department and psychiatric inpatient care, community- and hospital-based outpatient care, and online services; and (5) a suicidal behaviour component that captures self-harm hospitalisations (used as a proxy for suicide attempts—see the “Limitations” section) and suicide deaths.

**Fig. 1:**
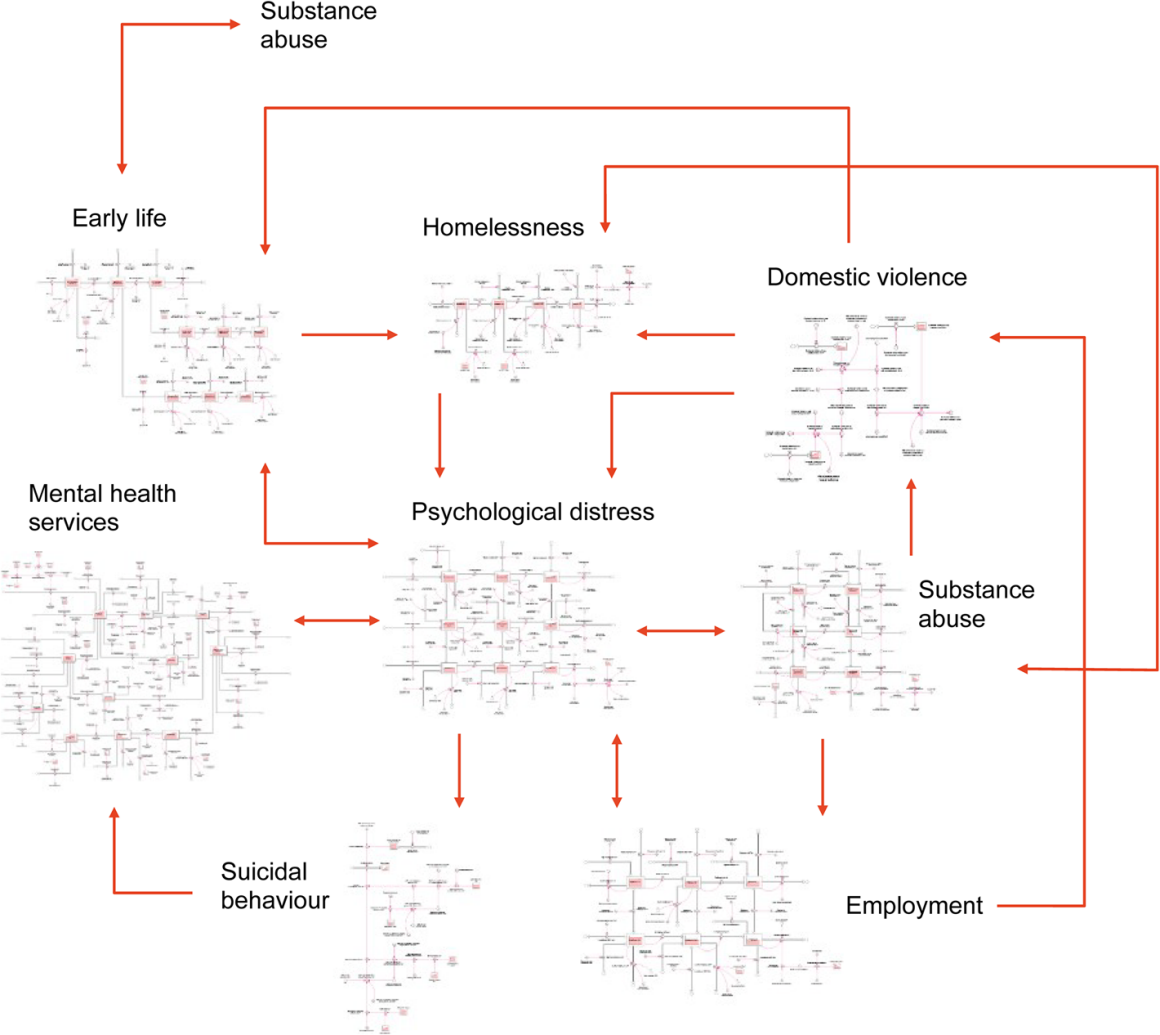
A high-level overview of the causal structure and pathways of the system dynamics model

Figure 1 provides a high-level overview of the causal structure and pathways of the model, with arrows denoting unidirectional or bidirectional relationships between each component. The structure and assumptions relating to each component and their interactions are detailed in Additional file 1. The model captures changes over time (dynamics) within each component and between the components of the model. For example, within the health system component, the proportion of the population waiting for services, receiving services, or disengaging from services changes over time based on service system capacity and the rates of flow into, within, and out of the service system. Dynamics also occur between the model components, for example, as unemployment rises, not only does it directly act to increase the incidence of high to very high psychological distress in the modelled population (which has flow-on effects on rates of substance misuse, and adverse early life exposures), but it also increases rates of domestic violence and homelessness, both of which further increase the rate psychological distress. Such dynamics makes it difficult to anticipate what impacts might occur across the system and on key outcomes if we intervene on one or multiple components of the model.

Primary model outputs included total (cumulative) numbers of self-harm hospitalisations and suicide deaths for the youth population (15–24 years) and total population. The model also provided estimates of the prevalence of low, moderate, and high to very high psychological distress by age categories (15–24 years, 25–64 years, 65 years, and above) and a range of measures of mental health service usage (e.g. mental health-related general practice consultations, psychiatric and allied mental health service consultations, services waiting times, numbers of psychologically distressed consumers that have disengaged from services, Emergency Department (ED) presentations, and psychiatric hospitalisations). All outputs were calculated every 0.4375 days (i.e. one sixteenth of a week) over a period of 30 years, starting from 1 January 2011, permitting comparisons of model outputs with historic data from 2011 to 2017 (see Additional file 1) and forecasts of the impacts of intervention scenarios described below simulated from the time of implementation (2021) to the start of 2041. This longer-term forecast horizon was deemed necessary to allow impacts of variations in the social determinants of mental health to be seen, as well as to encourage transition to a long-term strategic outlook in assessing the value of investment decisions rather than the current short-term perspective that induces more reactive decision making.

Parameter values that could not be derived directly from available data or published research were estimated via constrained optimisation, using historical time series data on a wide range of mental health and social outcomes, including psychological distress prevalence, self-harm hospitalisation and suicide rates, rates of mental health services usage (general practice consultations, specialised psychiatric services, ED and hospital inpatient care, community-based mental health services), substance abuse disorder prevalence, unemployment and labour force participation rates, domestic violence incidence, and the prevalence of homelessness. Powell’s method [51] was employed to obtain the set of (optimal) parameter values minimising the sum of the mean absolute percent error calculated for each time series separately (i.e. the mean of the absolute differences between the observed time series values and the corresponding model outputs, where each difference is expressed as a percentage of the observed value).

### Policy testing and sensitivity analyses

We modelled the potential impacts on suicidal behaviour of a set of interventions identified by the PHN and stakeholder group as most relevant to the North Coast NSW context. Interventions were identified based on alignment with current investment priorities and feasibility of implementation and included (1) six mental health and suicide prevention and (2) five social determinants modifiers. Details of each intervention (and assumptions) are provided in Additional file 1: Table S1. Twenty-one alternative scenarios (Table 1) were compared with a baseline (business as usual), in which existing policies and programs remain in place and current per capita growth in mental health services capacity is maintained until the end of the simulation.

**Table 1:** Scenarios examined in the simulation analyses (additional details are provided in Additional file 1)

Intervention	Description
1. Mental health and suicide prevention interventions	
a. Post-attempt assertive aftercare	Post-attempt assertive aftercare is an active outreach and enhanced contact program to reduce readmission in those presenting to services after a suicide attempt. It includes individually tailored contact, solution focused counselling, and motivations to adherence to follow-up treatments and continuity of contact.
b. Social connectedness programs	Programs designed to increase community connectedness, reducing isolation, and enhancing resilience and applied universally. No assumptions are made about the details of the particular programs implemented as these are community designed and are likely to differ across communities.
c. Community-based acute care services	Responsive clinical mental health services delivered by community mental health teams. People in suicidal crisis may call and request either a home-based visit or a centre-based visit, depending on their level of functioning and risk.
d. Technology-enabled crisis response	Responsive clinical mental health services delivered by community mental health teams. People in suicidal crisis may call and request either a home-based visit or a centre-based visit, depending on their level of functioning and risk.
e. Technology-enabled coordinated care	Technology-enabled coordinated care involves the use of online technology to facilitate delivery of multidisciplinary team-based care, in which medical and allied health professionals consider all relevant treatment options and collaboratively develop an individual treatment and care plan for each patient.
f. Post-discharge peer support	Post-discharge peer support is based on the Hospital to Home (H2H) program. This intervention involves peer workers (i.e. individuals with their own lived experience of mental illness and recovery) providing individualised practical and emotional support to patients discharged from psychiatric hospital care.
g. Post-attempt care PLUS Social connectedness PLUS Technology-enabled coordinated care	Interventions a, b, and e combined.
h. All mental health and suicide prevention interventions	Interventions a, b, c, d, e, and f combined.
2. Social determinants	
i. Reducing childhood adversity by 20%	Reduces the rates at which children (aged 0–14 years) at low and moderate risk of developing a mental disorder transition to moderate and high levels of risk by 20%
j. Reducing childhood adversity by 50%	Reduces the rates at which children (aged 0–14 years) at low and moderate risk of developing a mental disorder transition to moderate and high levels of risk by 50%
k. Increasing youth employment by 20%	Increases the rate at which unemployed young people (aged 15–24 years) secure employment by 20%
l. Increasing youth employment by 50%	Increases the rate at which unemployed young people (aged 15–24 years) secure employment by 50%
m. Reducing (total) unemployment by 20%	Reduces the age-specific rates at which employed people (aged 15 years or more) become unemployed by 20%.
n. Reducing (total) unemployment by 50%	Reduces the age-specific rates at which employed people (aged 15 years or more) become unemployed by 50%.
o. Reducing domestic violence by 20%	Reduces domestic violence rates (incidents reported per year) among people aged 15 years and above by 20%
p. Reducing domestic violence by 50%	Reduces domestic violence rates (incidents reported per year) among people aged 15 years and above by 50%
q. Reducing homelessness by 20%	Reduces age-specific rates at which people in secure housing enter homelessness by 20%
r. Reducing homelessness by 50%	Reduces age-specific rates at which people in secure housing enter homelessness by 50%
s. Reducing childhood adversity by 50% PLUS increasing youth employment by 50%	Scenarios j and l combined.
t. All social determinants in combination	Scenarios j, l, n, p, and r combined.
u. Best combination of mental health and suicide prevention interventions PLUS best combination of social determinants	Scenarios g and s combined.

Sensitivity analyses were performed to assess the impact of uncertainty in estimates of the direct effects of each intervention and forecasted growth in services capacity (i.e. GP mental health services, psychiatrists and allied services, community mental health services, psychiatric hospital care, and alcohol and drug services) on the simulation results. We used Latin hypercube sampling to draw 100 sets of values for the selected model parameters from a uniform joint distribution spanning ± 20% of the default values (see Additional file 1: Table S2). Differences in projected (cumulative) numbers of self-harm hospitalisations and suicide deaths between the baseline and intervention scenarios were calculated for each set of parameter values and summarised using simple descriptive statistics. This research was approved by the University of Sydney Human Research Ethics Committee (Project number: 2018/833).

## Results

**Fig. 2:**
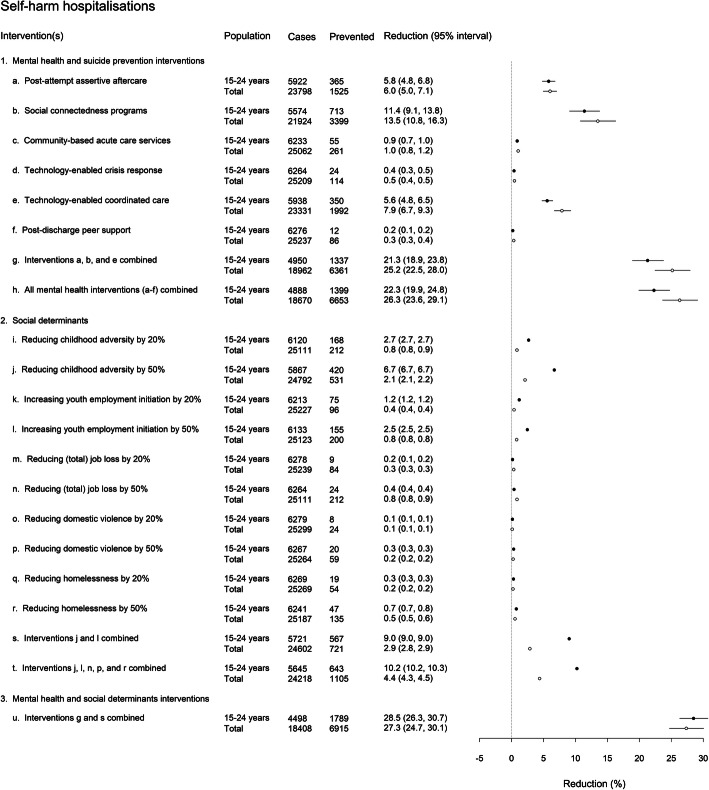
Differences in projected total (cumulative) numbers of self-harm hospitalisations between baseline and intervention scenarios (2021–2041). Numbers of cases (i.e. hospitalisations) and cases prevented are rounded to the nearest integer and were obtained assuming the default parameter values. Mean percentage reductions and 95% intervals reported in the rightmost column and plotted on the right were derived from the distributions of projected outcomes calculated in the sensitivity analyses (note that the 95% intervals provide a measure of the impact of uncertainty in the assumed intervention effects but should not be interpreted as confidence intervals)

**Fig. 3:**
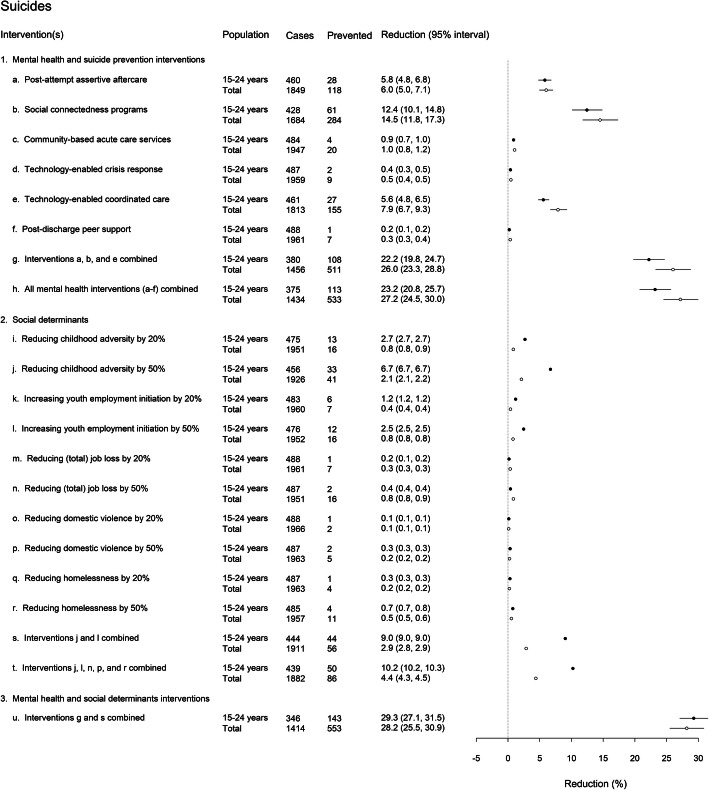
Differences in projected total (cumulative) numbers of suicides between the baseline and intervention scenarios (2021–2041). Numbers of cases (i.e. suicides) and cases prevented are rounded to the nearest integer and were obtained assuming the default parameter values. Mean percentage reductions and 95% intervals reported in the rightmost column and plotted on the right were derived from the distributions of projected outcomes calculated in the sensitivity analyses (note that the 95% intervals provide a measure of the impact of uncertainty in the assumed intervention effects but should not be interpreted as confidence intervals)

Rates of suicide are projected to slightly decrease over the forecast period (2021–2041) with the suicide rate for the total population decreasing from 18.7 to 15.1 per 100,000 population per year and for the youth population decreasing from 47.8 to 42.7 per 100,000 population per year. Under the baseline scenario, approximately 25,360 self-harm hospitalisations and 1970 suicide deaths were forecast over the period (1 January 2021 to the end of 2041), which includes 6292 self-harm hospitalisations and 489 suicide deaths in young people aged 15–24 years. Reductions in the numbers of self-harm hospitalisations and suicide deaths relative to these baseline estimates for each intervention scenario are presented in Figs. 2 and 3 with uncertainty intervals.

### Projected impacts of a range of locally prioritised mental health and suicide prevention interventions

**Fig. 4:**
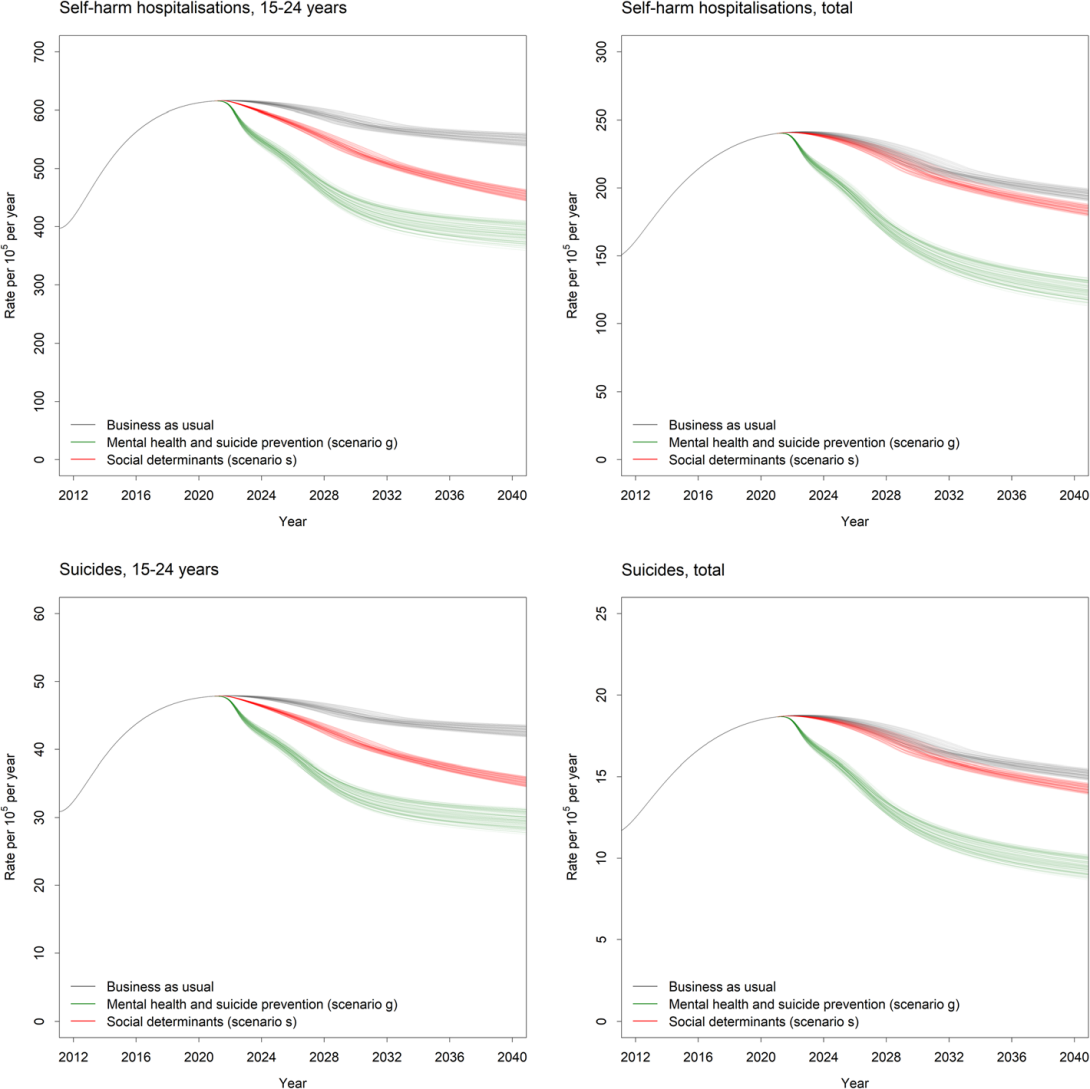
Self-harm hospitalisation and suicide rates (per 10^5^ population per year). Projections are shown for the baseline scenario (i.e. business as usual) and for selected combinations of mental health and suicide prevention interventions (post-attempt care plus social connectedness programs plus technology-enabled coordinated care; scenario g in Figs. 2 and 3) and social determinants interventions (a 50% reduction in childhood adversity plus a 50% increase in youth employment initiation; scenarios in Figs. 2 and 3)

Programs effective in increasing social connectedness had the single greatest impact on suicidal behaviour in the youth population and total population, reducing the numbers of self-harm hospitalisations by 11.4% and 13.5%, respectively, and suicide deaths by 12.4% and 14.5% respectively. Relatively large reductions were also forecasted with the implementation of technology-enabled coordinated care (reducing self-harm hospitalisations and suicide deaths by 5.6% each), and post-attempt assertive aftercare (reducing self-harm hospitalisations and suicide deaths by 5.8% each) in the youth population, with slightly higher reductions across the total population. The combined impact of these three interventions were forecast to reduce self-harm hospitalisation by 21.3% and suicide deaths by 22.2% in the youth population with relatively rapid reductions over the first decade that plateau over the second decade (Fig. 4). Implementing all the specific mental health and suicide prevention interventions failed to achieve substantial additional reductions in suicidal behaviour across the total or youth populations than achieved through the ‘best combination.’

### Projected impacts of combining specific mental health and suicide prevention interventions

The model projected a substantial reduction in self-harm hospitalisations (28.5%) and suicide deaths (29.3%) in a youth population by combining social connectedness programs, technology-enabled coordinated care, and post-attempt assertive aftercare with a reduction in childhood adversity by 50% and an increase in youth employment initiation by 50%. Similar impacts were achieved across the total population with this combination.

### Projected impacts of investments across the social determinants of mental health in the region

Of the scenarios targeted at reducing the social determinants of mental health, reducing childhood adversity by 50% had the single greatest impact on suicidal behaviour in the youth population, reducing the numbers of self-harm hospitalisations and suicide deaths by 6.7% each. Combining reductions in childhood adversity with a 50% increase in youth employment initiation is forecast to deliver reductions in self-harm hospitalisations and suicide deaths of 9.0% each and an effect that is amplified over time (Fig. 4). The impact of this combination on the total population is far less reducing self-harm hospitalisations and suicide deaths by 2.9% each. Implementing all scenarios across the social determinants of mental health did not produce substantial additional benefits in reducing suicidal behaviour across the total or youth populations than achieved through the ‘best combination.’

## Discussion

This study used systems modelling and simulation to leverage a range of national, state, and local data sets and best evidence and undertake a priori testing of scenarios that explore the impact on suicide of investing across social determinants of mental health and specific mental health and suicide prevention initiatives. Of the intervention scenarios examined, improving social connectedness was the single most effective intervention in reducing SB across the youth and total populations over the long term. This finding is consistent with studies highlighting social isolation as an important contributing factor to suicide [52, 53]. While only the broad strategy of community support programs aimed at increasing social connectedness and reducing social isolation was modelled, the specific features of these programs were not prescribed in recognition of the vital importance of engaging local communities in their design and delivery within a cultural framework of community development [54, 55]. However, locally designed and implemented programs should be evaluated to facilitate future model refinement.

Findings of this study also demonstrated that improvements to the social determinants of mental health do not contribute equally to reductions in suicidal behaviour. Investments in reducing childhood adversity and increasing youth employment initiation together represent best targets, not only for their impact over the selected time horizon, but also for their ongoing amplifying effects in reducing youth suicide over the longer term. These findings are consistent with reviews of the literature of individual and ecological studies highlighting the strong associations that youth unemployment and exposure to early life adversity (particularly sexual abuse and accumulation of adversities) have on youth suicidal behaviour [56, 57] and point to the importance of looking to broader social, educational and vocational targets for the prevention of SB [58]. While these findings highlight early life exposures and youth unemployment as important potential targets for investments for youth suicide prevention, they do not suggest a lack of importance for addressing adult unemployment, domestic violence, and homelessness on broader moral, social, and economic grounds.

While no specific programs were modelled, reducing childhood adversity by between 20 and 50% was projected to deliver the single greatest impact on suicide rates in young people among the social determinants, suggesting it to be a worthwhile target for investment and action. The scenario of a reduction in childhood adversity was modelled by multiplying the rates at which children (aged 0–14 years) at low and moderate risk of developing a mental disorder transition to moderate and high levels of risk. Therefore, programs to reduce childhood adversity may be targeted at either primary prevention (i.e. strategies to reduce exposure to domestic and family violence, abuse, neglect, poverty, war, and natural disasters) [59–61] and/or at programs aimed at harm minimisation to reduce the risk of emergence of mental disorders among children and adolescence that have experienced adversity. Harm minimisation may include screening, referral, and intervention carried out in clinical settings [62] and/or the implementation of universal, school- or community-based, resilience-focused interventions to provide more generalised fostering of mental health [63]. Determining the feasibility and nature, targeting, timing, scale, and duration of programs needed to achieve a 20% or 50% reduction in childhood adversity are best explored in partnership with regional communities through extension of the existing systems model to test alternative strategies prior to implementation, monitoring, and evaluation of programs.

Finally, there are two important insights that this systems modelling study highlights. Firstly, that the greatest impacts on suicidal behaviour in young people are likely to be achieved with a mix of specific mental health and suicide prevention initiatives (with more immediate impacts that plateau over time) and improvements to key social determinants (with delayed impacts but amplifying effects over the longer term) as highlighted in Fig. 4. Secondly, this study highlights that more is not necessarily better. The simulated impacts of implementing all mental health and suicide prevention initiatives included in the model were little better than the impacts of the targeted combination of social connectedness programs, post suicide attempt assertive aftercare, and technology-enabled coordinated mental health care. This highlights the importance of the advanced decision support capability provided by systems modelling to facilitate a more strategic approach to the allocation of limited resources.

### Limitations

There are a number of limitations that require consideration when interpreting the findings of this study. There is potential measurement bias in the range of secondary data used to parameterise the model including the population health surveys, Medicare claims data, and PHN and Local Health District (LHD) datasets. The model acknowledges these potential sources of measurement bias and a number of commonly used strategies were employed to address them, including the triangulation of multiple data sources, parameter estimation via constrained optimisation, and local verification to identify plausible estimates.

In addition, there is potentially an under-enumeration of suicide cases used to calibrate the model, due to the misclassification of suicides to ICD codes relating to unintentional injury and events of ‘undetermined intent’ [64]. Suicide attempts identified from hospital admissions data likely only capture those cases serious enough to warrant medical intervention, and instances of self-harm where the intent was not clear may be not coded as suicide attempts. However, this under-enumeration is consistent across simulations of the baseline case and intervention scenarios and as such are unlikely to affect the forecast estimates of impact (i.e. the % reduction in suicidal behaviour) of intervention strategies or the strategic insights derived from the model. Ongoing systematic monitoring and evaluation can determine the extent to which the model forecasts are corresponding with real-world outcomes over time, allowing refinement of model parameters to improve forecasting capabilities. Finally, as the impacts of simulated scenarios are subject to the population, demographic, behavioural, and service dynamics of the modelled region, they are not necessarily generalisable to other regions; however, depending on contextual similarity to the modelled region, the qualitative insights are likely to relevant.

## Conclusions

The findings of this study suggest that targeted investments in addressing the social determinants and in mental health services provides the best opportunity to reduce SB and suicide. The current prioritised set of findings are by no means intended to provide ‘the answer’, but rather to demonstrate how systems models can bring together a body of evidence and data in way that facilitates learning among system actors regarding system behaviour in response to the introduction of new initiatives and ‘solutions’. This systems model is providing regional decision makers and stakeholders the capacity to investigate alternative scenarios related to the timing of implementation of interventions, their scale and intensity, and to test alternative assumptions regarding level of intervention uptake to inform strategic decision making. The potential of systems modelling and simulation to support a more disciplined, targeted, inclusive, and transparent approach to national and regional decision-making regarding allocation of resources to reduce suicidal behaviour has been well described [12, 29, 34]. Importantly, such interactive systems models are being used to explore the impact of possible investment decisions on outcomes other than suicidal behaviour to ensure that unintended negative impacts on other parts of the system, such as mental health ED presentations, service wait times, and capacity, do not occur as a result of efforts to specifically address suicidal behaviour.

Finally, it is important to note that this study focussed on simulations of *improvements to* the social determinants of mental health in the North Coast NSW region, which may underestimate their importance in comparison to the specific mental health and suicide prevention interventions simulated to reducing suicidal behaviour. Inevitable corollaries of the COVID-19 pandemic are a *deterioration in* the social determinants of mental health, which may produce greater negative impacts on suicidal behaviour than the positive impacts of improvements to those determinants simulated in the current study. As shown in other applications of systems modelling in mental health [38], important thresholds might exist where deterioration in current levels of unemployment in the North Coast NSW population catchment may result in greater than anticipated increases in substance misuse and suicidal behaviour. This is the subject of further investigation.

## Supplementary Information

**Additional file 1.** Detailed model structure, parameter estimates, and other numerical inputs of the North Coast PHN system dynamics model for suicide prevention and mental health services planning.

## Data Availability

The datasets generated and analysed during the current study are available from the corresponding author on reasonable request.
